# Melanoma recurrence patterns and management after adjuvant targeted therapy: a multicentre analysis

**DOI:** 10.1038/s41416-020-01121-y

**Published:** 2020-10-22

**Authors:** Prachi Bhave, Lalit Pallan, Georgina V. Long, Alexander M. Menzies, Victoria Atkinson, Justine V. Cohen, Ryan J. Sullivan, Vanna Chiarion-Sileni, Marta Nyakas, Katharina Kahler, Axel Hauschild, Ruth Plummer, Claudia Trojaniello, Paolo A. Ascierto, Lisa Zimmer, Dirk Schadendorf, Clara Allayous, Celeste Lebbe, Andrea Maurichi, Mario Santinami, Severine Roy, Caroline Robert, Thierry Lesimple, Sapna Patel, Judith M. Versluis, Christian U. Blank, Adnan Khattak, Andre Van der Westhuizen, Matteo S. Carlino, Mark Shackleton, Andrew Haydon

**Affiliations:** 1 Department of Medical Oncology, Alfred Hospital, Melbourne, VIC Australia; 2 Melanoma Institute Australia, The University of Sydney, Sydney, NSW Australia; 3 Department of Medical Oncology, Royal North Shore Hospital, Sydney, NSW Australia; 4 Department of Medical Oncology, Princess Alexandra Hospital, Greenslopes Private Hospital and University of Queensland, Brisbane, QLD Australia; 5 Department of Medical Oncology, Massachusetts General Hospital, Boston, MA USA; 6 Melanoma Oncology Unit, Veneto Institute of Oncology-IRCCS, Padova, Italy; 7 Department of Oncology, Oslo University Hospital, Oslo, Norway; 8 Department of Dermatology, University Hospital Schleswig-Holstein, Campus Kiel, Kiel, Germany; 9 Northern Centre for Cancer Care, Freeman Hospital, Newcastle upon Tyne, UK; 10 Department of Melanoma and Cancer Immunotherapy, Istituto Nazionale Tumori IRCCS Fondazione Pascale, Napoli, Italy; 11 Department of Dermatology, University Hospital Essen, Essen & German Cancer Consortium, Heidelberg, Germany; 12 AP-HP Dermatology Department, Saint-Louis Hospital, Paris, France; 13 Department of Surgery, Fondazione IRCCS Istituto Nazionale dei Tumori di Milano, Milan, Italy; 14 Department of Dermatology, Gustave Roussy and Paris-Saclay Institute, Villejuif, France; 15 Department of Medical Oncology, Centre Eugène Marqui, Rennes, France; 16 Department of Melanoma Medical Oncology, MD Anderson Cancer Centre, Houston, TX USA; 17 Department of Medical Oncology, Netherlands Cancer Institute, Amsterdam, The Netherlands; 18 Department of Medical Oncology, Fiona Stanley Hospital, Perth, WA Australia; 19 Department of Medical Oncology, Calvary Mater Hospital, Newcastle, NSW Australia; 20 Department of Medical Oncology, Westmead Hospital, Sydney, NSW Australia; 21 Central Clinical School, Monash University, Melbourne, VIC Australia

**Keywords:** Targeted therapies, Melanoma, Melanoma

## Abstract

**Background:**

Adjuvant targeted therapy (TT) improves relapse free survival in patients with resected BRAF mutant stage III melanoma. The outcomes and optimal management of patients who relapse after adjuvant TT is unknown.

**Methods:**

Patients from twenty-one centres with recurrent melanoma after adjuvant TT were included. Disease characteristics, adjuvant therapy, recurrence, treatment at relapse and outcomes were examined.

**Results:**

Eighty-five patients developed recurrent melanoma; nineteen (22%) during adjuvant TT. Median time to first recurrence was 18 months and median follow-up from first recurrence was 31 months. Fifty-eight (68%) patients received immunotherapy (IT) or TT as 1st line systemic therapy at either first or subsequent recurrence and had disease that was assessable for response. Response to anti-PD-1 (±trial agent), combination ipilimumab-nivolumab, TT rechallenge and ipilimumab monotherapy was 63%, 62% 25% and 10% respectively. Twenty-eight (33%) patients had died at census, all from melanoma. Two-year OS was 84% for anti-PD-1 therapy (±trial agent), 92% for combination ipilimumab and nivolumab, 49% for TT and 45% for ipilimumab monotherapy (*p* = 0.028).

**Conclusions:**

Patients who relapse after adjuvant TT respond well to subsequent anti-PD-1 based therapy and have outcomes similar to those seen when first line anti-PD-1 therapy is used in stage IV melanoma.

## Background

The management of cutaneous melanoma has been revolutionised in the last decade. Targeted therapies (TT) inhibiting the MAPK pathway and immunotherapy (IT) with T-cell checkpoint inhibitors have each been demonstrated to prolong survival of patients with metastatic melanoma.^1–5^ As a result, these therapies are now mainstays of treatment for patients with unresectable stage III or stage IV disease.

Recent studies have tested adjuvant systemic TT and IT for resected stage III/IV melanoma, with the aims of reducing melanoma recurrence and prolonging patient survival.^6,7^ Whilst TT is reserved for patients with BRAF _V600_ mutant melanoma, IT may be used in patients irrespective of their BRAF status.

A number of randomised, Phase 3 trials have investigated IT as adjuvant treatment in melanoma. The EORTC-18071 trial compared adjuvant ipilimumab 10 mg/kg to placebo in patients with resected stage III disease and demonstrated an improvement in both relapse free survival (RFS, hazard ratio (HR) 0.76, *p* = 0.0008) and overall survival (OS) (HR 0.72, *p* = 0.001). However, toxicity rates were high, with 45% of patients having grade 3–4 adverse events (AEs), resulting in a third of patients discontinuing treatment.^8–10^ The Phase 3 E1609 trial examined the use of adjuvant ipilimumab at two doses (3 mg/kg and 10 mg/kg) and interferon alfa-2b. The lower ipilimumab dose improved OS compared to interferon, however, rates of grade 3–4 AEs were not negligible, and therefore single agent ipilimumab is rarely used in clinical practice.^11^

The Checkmate-238 trial randomised patients with resected stage IIIB/C or IV disease to nivolumab 3 mg/kg or ipilimumab 10 mg/kg for 1 year, finding an improvement in RFS (HR 0.66, *p* < 0.0001) for nivolumab over ipilimumab, with only 14% of nivolumab patients having grade 3–4 AEs.^12,13^ Discontinuation of nivolumab due to grade 3–4 AEs occurred in 4.6% of patients, whereas 31% of ipilimumab patients discontinued treatment. Keynote-054 randomised patients with resected stage III melanoma to either pembrolizumab or placebo, also demonstrating an improvement in RFS with pembrolizumab (HR 0.57, *p* < 0.001). Toxicity from pembrolizumab was comparable to that of nivolumab in Checkmate-238, with 14% of patients suffering a grade 3–4 adverse event.^14^ Of note, EORTC-18071 and Keynote-054 excluded patients with <1 mm of micro-metastatic disease in lymph nodes and all three adjuvant IT trials used the 7th edition of the AJCC melanoma staging system.

Two trials have examined adjuvant TT in BRAF mutant patients. The BRIM-8 trial included two cohorts of patients with resected melanoma (Cohort 1: stage IIC, IIIA, IIIB and Cohort 2: stage IIIC) that were randomised to single agent vemurafenib or placebo. A two-cohort design was implemented to prevent the risk of stage IIIC patients driving the analysis due to their worse prognosis. The trial’s primary end point of disease-free survival (DFS) underwent prespecified hierarchical testing, wherein DFS was tested in Cohort 2 before Cohort 1. After one year of adjuvant vemurafenib, the trial did not meet statistical significance in Cohort 2, thereby rendering Cohort 1’s data also statistically insignificant.^15^ As a result, the use of adjuvant single agent BRAF inhibitors is not recommended.

The COMBI-AD trial tested dual agent dabrafenib and trametinib (DT) adjuvantly for one year in patients with resected stage III disease. The trial met its primary end point of RFS. At 4-years of follow-up, the RFS rate was 54% in the adjuvant DT arm, compared to 38% in the placebo arm, giving a HR of 0.49.^16,17^ An interim analysis at three years demonstrated an OS of 86% with adjuvant DT compared to 77% for placebo (HR 0.57, 95% CI 0.42–0.79, *p* = 0.0006) although the *p*-value did not meet the prespecified significance boundary of 0.00019. Final OS data is not yet available as the number of prespecified events has not yet been reached. Toxicity associated with adjuvant DT was not insignificant, with 31% of patients experiencing grade 3–4 AEs due to DT, compared to 5% in the placebo group. Furthermore, 26% of patients prematurely ceased adjuvant DT due to toxicity; although, importantly, there were no treatment related deaths. As a result of this trial, dual agent TT has become a standard treatment option for adjuvant therapy in patients with fully resected stage III BRAF mutant melanoma.

Although these trials have transformed treatment for stage III melanoma, the outcomes of patients who relapse after adjuvant therapy are not well described. For example, although current retrospective data suggest that response rates (RRs) to TT are high in patients who relapse with unresectable disease after adjuvant IT,^18^ little is known about the outcomes of patients who relapse after adjuvant TT, including their rates of response to subsequent lines of therapy and survival. We sought to address this in a multi-centre, international cohort analysis.

## Methods

### Institutional ethics board approval was obtained for the analysis

Patients with resected stage III or IV melanoma (as per AJCC 8th edition) who recurred after receiving single or dual agent adjuvant TT were included in this study.

Data was extracted from twenty-one melanoma centres worldwide. Patients were included by searching electronic and paper hospital records and institutional databases to identify patients enrolled in adjuvant TT trials (BRIM-8 or COMBI-AD), those who accessed adjuvant TT through patient access programs, as well as from hospital pharmacy records. If patients had participated in a randomised trial involving placebo, they were included if they had been unblinded at the time of melanoma recurrence and confirmed to have received adjuvant TT.

Patient records were retrospectively analysed and data on patient demographics were collected including age at diagnosis of resected stage III or IV melanoma and baseline melanoma characteristics such as tumour thickness, primary site, presence or absence of ulceration, mitotic rate and type of BRAF mutation. Information was recorded on diagnosis of stage III or IV melanoma using AJCC 8^th^ edition staging, on performance of completion lymph node dissection (CLND) and on the number of lymph nodes with metastatic disease. Details on adjuvant TT were noted, including type used, duration and reason for cessation of therapy. Information was recorded on melanoma recurrence, including stage at recurrence, method of detection of recurrence, and sites of recurrent disease. Subsequent therapy was recorded, including modality of treatment, duration, response and complications. Imaging with CT of the chest, abdomen and pelvis or PET scan in combination with either a CT brain or MRI brain was performed. For patients on a clinical trial, imaging frequency occurred as per trial protocol; for patients not on trial, imaging occurred as per standard of care at each institution. Tumour response was evaluated by investigator review: any degree of tumour shrinkage was deemed a partial response, disease control was recorded as stable disease, resolution of all disease on CT or complete metabolic response on PET was considered a complete response and increase in tumour size or clinical deterioration due to melanoma was deemed progressive disease. Patients were followed until death or the data censorship date.

The Kaplan–Meier method was used to create survival curves. Comparisons between survival curves were made using the Log rank test. Cox regression was used to perform the multivariate analysis. All statistical analyses were performed using SPSS version 22.0.

## Results

### Patient characteristics

Eighty-seven patients from centres across Australia, Europe and the United States of America met inclusion criteria. Two patients were excluded due to prolonged duration of adjuvant TT (one for over 3 years and one for 1 year 8 months). Thus, the final analysis included 85 patients. Data was collected from January 2013 to September 2019.

Baseline patient characteristics are summarised in Table 1. The study population had a slight male predominance of 56% (*n* = 48). Median age at time of diagnosis of resected stage III or IV melanoma was 47 years (range 22–74 years). The majority (*n* = 81, 95%) of patients had primary cutaneous melanoma, with four (5%) having no identifiable primary skin lesion (occult melanoma). 88% (*n* = 75) of patients had a BRAF V600E mutation whilst 7% (*n* = 6) had a confirmed BRAF V600 mutation without subtyping. Of the included patients, 48% (*n* = 41) were from Europe, with the remainder from Australia (*n* = 36, 42%) or the United States of America (*n* = 8, 9%). All patients were staged according to the 8^th^ edition of the AJCC staging system, with 61% of patients having stage IIIC disease and 2% having resected stage IV disease.

**Table 1:** Baseline patient characteristics; melanoma staging as per AJCC 8th edition.

Characteristics	Patient number, *N* = 85 (%)
Sex–no. (%)	
Male	48 (56)
Female	37 (44)
Median age at diagnosis of resected stage III or IV-year (range)	47 (22–74)
Primary Site–no. (%)	
Cutaneous	81 (95)
Occult	4 (5)
BRAF mutation–no. (%)	
V600E	75 (88)
V600K	3 (4)
V600R	1 (1)
V600^a^ (unspecified)	6 (7)
Patient origin–no. (%)	
Australia	36 (42)
Europe	41 (48)
USA	8 (9)
Melanoma Stage at Adjuvant Treatment (AJCC 8th edition)–no. (%)	
Stage III	83 (98)
IIIA	5 (6)
IIIB	21 (25)
IIIC	52 (61)
IIID	5 (6)
Resected stage IV	2 (2)

*AJCC* American Joint Committee on Cancer, *USA* United States of America.

^a^Six patients, all from a single centre in Europe, had a known BRAF V600 mutation but subtype was unknown.

### Adjuvant therapy

Details on adjuvant TT received are summarised in Supplement 1. Eighty-one (95%) patients had undergone complete lymph node dissection prior to commencement of adjuvant TT. Seventy-three (86%) patients received adjuvant DT, one (1%) received adjuvant vemurafenib and cobimetinib combination and eleven (13%) received single agent vemurafenib. Four (5%) patients received adjuvant radiotherapy (RT) before adjuvant TT, prior to first recurrence. Fifteen (18%) patients received neoadjuvant followed by adjuvant TT. Median duration of adjuvant therapy was 8.6 months. The majority (53%) of patients ceased adjuvant TT due to completion of the prescribed course.

### Disease characteristics at first recurrence

Melanoma and patient characteristics at first recurrence after adjuvant TT are summarised in Table 2. Nineteen (22%) patients recurred during adjuvant TT and sixty-six (78%) after cessation of adjuvant TT. Twenty-nine (34%) patients recurred locoregionally, fifty (59%) patients had distant recurrence and six (7%) patients had both locoregional and distant recurrence.

**Table 2:** Disease characteristics at first recurrence including stage and method of detection of recurrence.

1st Recurrence characteristics	Patient number, *N* = 85 (%)
Median time to 1st recurrence- months (range)	17.7 (1.7–53.6)
ECOG at recurrence–no. (%)	
0	75 (88)
1	8 (9)
2	0 (0)
3	1 (1)
Unknown	1 (1)
Melanoma stage at 1st Recurrence (AJCC 8th edition)–no. (%)	
III	26 (31)
IIIB	3 (4)
IIIC	18 (21)
IIID	5 (6)
IV	53 (62)
M1a	14 (17)
M1b	12 (14)
M1c	13 (15)
M1d	14 (17)
Locoregional and distant recurrence	6 (7)
Recurrence primarily detected by–no. (%)	
Symptoms	10 (12)
Clinical examination	26 (31)
Imaging	49 (57)

The majority (88%) of patients had an ECOG of 0 at recurrence and 57% of recurrences were detected on imaging, in the absence of symptomatic or clinically apparent disease. Median time to first recurrence was 17.7 months (1.7–53.6) and median time to development of distant recurrence was 19.2 months (3.1–56.4).

### Treatment at first recurrence

At first recurrence, four (5%) patients received RT only, all with palliative intent. Twenty-one (25%) patients underwent surgery alone, of which 17 (17/21, 81%) had further recurrence of disease. Five (6%) patients underwent surgery and RT combined, and all of these recurred subsequently. Thus, 85% (22/26) of patients who underwent surgery without systemic therapy at first recurrence developed a second recurrence of melanoma. Of the four patients who did not recur after surgery for first recurrence, three had distant disease and one had local disease at first recurrence.

**Fig. 1:**
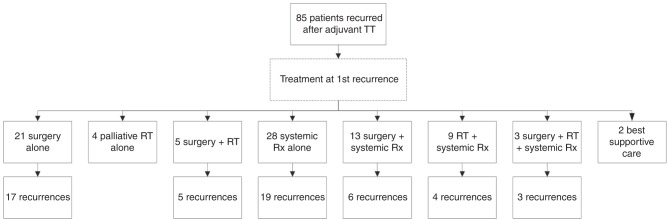
Flow chart of treatment at first recurrence of melanoma after adjuvant TT. Rx therapy, RT radiotherapy, TT targeted therapy.

Twenty-eight (33%) patients received systemic therapy alone at first recurrence, of which nineteen (19/28, 68%) developed a 2nd recurrence. Thirteen (15%) patients received surgery and systemic therapy at first recurrence, and six (6/13, 46%) of these developed a second recurrence. Nine (11%) patients received RT and systemic therapy at first recurrence of which four (4/9, 44%) subsequently recurred. Three (4%) received surgery, RT and systemic therapy combined; all (3/3, 100%) recurred again. Two (2%) patients received best supportive care (BSC) only (Fig. 1).

Of the 13 patients who underwent surgery and systemic therapy at first recurrence, 8 (8/13, 62%) patients had surgery to no evidence of disease (NED) followed by adjuvant anti-PD-1 based therapy. Of these patients, only 25% (2/8) developed a second recurrence of melanoma.

Of the 29 patients that recurred locoregionally at first recurrence, 23 (23/29, 79%) patients underwent surgery to NED and 14 patients later developed distant disease (four locoregional and distant recurrence, 10 distant recurrence only).

### Response to 1st line systemic therapy

Sixty-eight (80%) patients received TT or IT as 1^st^ line systemic therapy at either first or subsequent melanoma recurrence. Twenty-six (26/68, 38%) patients received anti-PD-1 based therapy, including single agent anti-PD-1 or anti-PD-1 in combination with an investigational agent. Fourteen (14/68, 21%) patients received ipilimumab and nivolumab combination immunotherapy. Twelve (12/68, 18%) patients received ipilimumab monotherapy and 16 (16/68, 24%) patients received TT. Of the 16 patients who received TT as 1st line systemic therapy after recurrence, only 5 (5/16, 31%) received drug alone, one of whom had a response (1 partial response (PR), 1 stable disease (SD), 3 progressive disease (PD)).

Response rate (RR) to 1st line systemic therapy after relapse was deemed assessable if patients received TT or IT at first or subsequent recurrence (with no prior systemic therapy) and had measurable disease for assessment. Two patients received adjuvant interferon at first recurrence followed by IT at second recurrence; these patients were also included in determining RR. Twenty-seven (32%) patients were not assessable for response: two received BSC, three received RT only for recurrence, five received surgery only, one underwent surgery at first recurrence then RT at second recurrence, five received non-IT or TT as 1st line systemic therapy (3 temozolomide, 1 interferon, 1 TVEC monotherapy), nine received surgery followed by adjuvant therapy (best response NED) and two patients completed treatment before disease assessment (both ipilimumab monotherapy).

Thus, 58 (68%) patients received IT or TT as 1st line systemic therapy at either first or subsequent recurrence and had disease that was assessable for response. RR to anti-PD-1 therapy, either as monotherapy or in combination with a trial agent was 63%, whilst RR to ipilimumab and nivolumab combination immunotherapy was 62%. RR to a rechallenge of TT was 25% and RR to single agent ipilimumab was 10% (Table 3). Of the 19 patients with evaluable disease who received anti-PD-1 based therapy, 6 (32%) were on a clinical trial involving an anti-PD-1 agent in combination with an investigational agent. Of these six patients, one had a complete response (CR) and 5 had a partial response (PR)- thus, 100% (6/6) had a response to anti-PD-1 and trial agent therapy. Of the thirteen patients who received anti-PD-1 monotherapy as 1st line systemic therapy, RR was 46% (6/13). Median time to first recurrence from start of adjuvant TT was 23 months for anti-PD-1 (±trial) therapy, 18 months for ipilimumab and nivolumab combination therapy, 13 months for TT rechallenge and 13 months for ipilimumab monotherapy.

**Table 3:** Response rates to 1st line systemic therapy after melanoma recurrence.

	Anti-PD-1 ± trial drug (*N* = 19)	Ipilimumab + Nivolumab (*N* = 13)	Targeted therapy (*N* = 16)	Ipilimumab single agent (*N* = 10)
Complete Response (CR)	4 (21%)	4 (31%)	1 (6%)	1 (10%)
Partial Response (PR)	8 (42%)	4 (31%)	3 (19%)	0
Stable Disease (SD)	0	0	1 (6%)	1 (10%)
Progressive Disease (PD)	7 (37%)	5 (38%)	11 (69%)	8 (80%)
Response Rate (RR)	63% (12/19)	62% (8/13)	25% (4/16)	10% (1/10)

Of the 21 patients who underwent surgery alone at first recurrence with no immediate adjuvant therapy, 9 (9/21, 43%) received subsequent anti-PD-1 based therapy as 1st line systemic therapy at second or subsequent relapse. Eight (8/9, 89%) of these patients responded to this subsequent anti-PD-1 therapy (5 CR, 3 PR).

### Overall survival

Median overall survival (mOS) from the date of first recurrence for all patients was not reached. Two-year OS for all patients from date of first recurrence was 71% (Supplement 2). Twenty-eight (33%) patients had died at census, all due to melanoma. Median follow-up from time of first recurrence was 31 months.

OS was not significantly different between patients who received single agent or combination TT as adjuvant therapy (*p* = 0.25).

There was also no significant difference in OS between patients who relapsed locoregionally or distally at first recurrence after adjuvant TT (Supplement 3; *p* = 0.16).

**Fig. 2:**
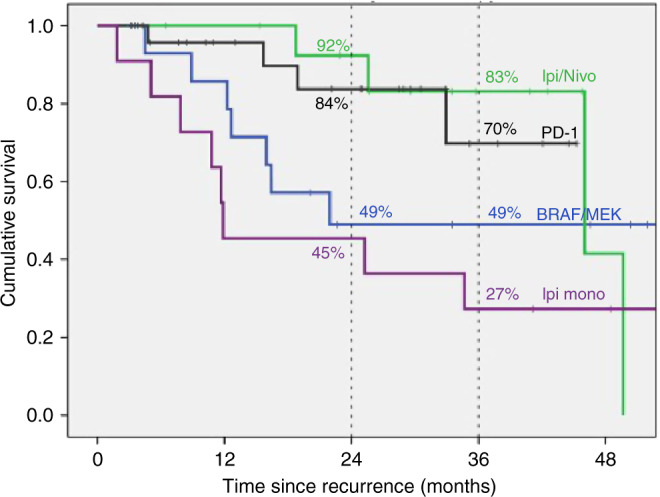
Kaplan–Meier curve of overall survival for all patients from time of first melanoma recurrence, by class of 1^st^ line systemic therapy received at recurrence (*p* = 0.028).

OS varied by drug class received as 1st line systemic therapy at relapse (Fig. 2). Two-year OS was 84% for anti-PD-1 therapy with or without a trial agent, 92% for ipilimumab and nivolumab combination immunotherapy, 49% for TT and 45% for ipilimumab monotherapy (*p* = 0.028). This remained significant in multivariant analysis for sex (*p* = 0.043).

### Recurrence during vs after adjuvant TT

**Fig. 3:**
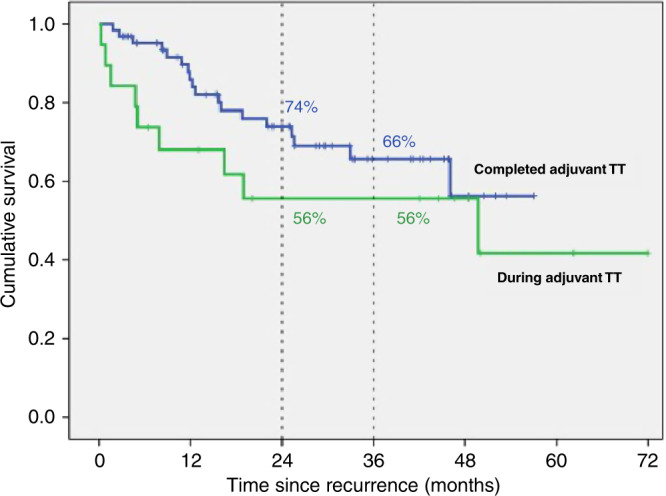
Kaplan–Meier curve of overall survival for patients who developed melanoma recurrence during and after adjuvant TT (*p* = 0.20).

No statistically significant difference in mOS occurred between patients who had recurrent melanoma whilst still receiving adjuvant TT compared to those who recurred after ceasing adjuvant TT (*p* = 0.20, Fig. 3).

## Discussion

The choice of adjuvant therapy for patients with BRAF mutant stage III melanoma is unclear, with compelling evidence supporting use of either TT or IT. Both approaches have led to similar relative risk reductions in RFS, and thus the decision between TT and IT is often dependant on patient and clinician preference. Many clinicians favour IT due to the sustained improvements in PFS and OS seen in the metastatic setting. It is important to note, however, that data is not yet available on whether adjuvant anti-PD-1 therapy leads to an improvement in OS. In contrast, COMBI-AD demonstrated an improvement in OS for adjuvant TT with a HR of 0.57 (*p* = 0.0006), though this did not reach the pre-specified significance boundary.

One factor of relevance when deciding between adjuvant TT and IT for BRAF mutant patients is knowledge of RRs and survival rates to subsequent systemic therapy upon relapse after adjuvant therapy. Owen *et al* presented retrospective data on patients who relapsed after adjuvant anti-PD-1 therapy, demonstrating that no patients responded to subsequent single agent anti-PD-1 if relapse occurred whilst on adjuvant anti-PD-1, whereas 40% of patients responded to a re-challenge of anti-PD-1 therapy if relapse occurred after cessation of adjuvant anti-PD-1. Interestingly, 79% of patients responded to TT if relapse occurred on adjuvant anti-PD-1, whilst 88% responded to TT after ceasing anti-PD-1 therapy.^18^ Thus, a change in treatment modality to either combination IT or TT is generally recommended in those patients that relapse on or after adjuvant single agent IT.

To our knowledge, this is the first study to report the RRs of BRAF mutant melanoma patients to subsequent systemic therapy following relapse after adjuvant TT. The RR of patients to anti-PD-1 based immunotherapy (anti-PD-1 with or without trial agent) after relapse was 63% and the RR to ipilimumab-nivolumab combination therapy was 62%, demonstrating that patients treated with adjuvant TT remain sensitive to IT. All patients (20/20, 100%) who responded to anti-PD-1 based IT as 1st line systemic treatment had developed recurrent melanoma after cessation of adjuvant TT. Of those patients who had completed adjuvant TT at recurrence, RR was 60% (6/10) to anti-PD-1 monotherapy (without a trial agent), 100% (6/6) for anti-PD-1 therapy with a trial agent and 73% (8/11) to ipilimumab-nivolumab combination treatment. These results compare favourably to RRs in the metastatic, treatment naive setting, with Checkmate-067 demonstrating objective RRs of 67% to ipilimumab-nivolumab combination and 37% to nivolumab monotherapy in BRAF mutant patients.^2^ Furthermore, patients treated with systemic therapy after relapse had a two-year OS rate of 92% for ipilimumab-nivolumab combination therapy and 45% for ipilimumab monotherapy. These results are also favourable when compared to those in Checkmate-067, where 2-year OS was 63% for ipilimumab- nivolumab and 43% for ipilimumab monotherapy.^2^

Of note, our study included two patients with resected stage IV melanoma, which varies from the cohort of patients included in most adjuvant trials. The COMBI-AD, EORTC 18071 and Keynote-054 trials included patients with resected stage IIIA-C disease, with resected stage IV patients being excluded. Checkmate-238 is the only key adjuvant trial to have included resected stage IV patients, though this trial involved adjuvant IT not TT. Despite our study including two patients with resected stage IV disease, these patients would not have influenced overall results as neither was assessable for response to 1st line systemic therapy at recurrence- one patient underwent resection of metastatic disease followed by adjuvant TT (best response NED) and the second patient received TVEC monotherapy.

In patients with metastatic melanoma, response to 2nd line single agent anti-PD-1 after 1st line palliative TT is ~25%.^19^ This is significantly less than the RR of 37% seen in the 1st line setting in BRAF mutant patients. This reduced efficacy after prior TT is why some clinicians advocate for IT to be given upfront in the metastatic setting, and this reasoning is often extrapolated to the adjuvant space. However, our study suggests that the RR to IT after prior adjuvant TT is not diminished when compared to 1st line IT in the metastatic setting. Most patients who are treated with TT in the metastatic setting are treated continuously until disease progression and are therefore truly resistant to BRAF/MEK therapy. In contrast, patients treated with adjuvant TT infrequently relapse during treatment, with most relapses occurring after completion of therapy. Interestingly, in our study, no patients who developed recurrence whilst receiving adjuvant TT responded to subsequent anti-PD-1 (±trial agent) or ipilimumab-nivolumab combination therapy, though only five patients met this criterion (three patients had PD to anti-PD-1 ± trial; two patients had PD to ipilimumab-nivolumab; no patients had SD to either agent). This lends support to the notion that at relapse, the biology and immunogenicity of disease that recurs after completion of TT may be different to disease that progresses during TT.^20–24^

In our cohort, 21% of patients ceased adjuvant TT due to toxicity, similar to the 26% discontinuation rate due to AEs seen in COMBI-AD, noting that our cohort also included single agent TT.^16^ Also, 22% developed recurrent melanoma while receiving adjuvant TT. Of note, in our study, patients were specifically included only if they had developed recurrent melanoma. Interestingly, no statistically significant difference in OS occurred between patients relapsing during adjuvant TT or after completion of adjuvant TT (*p* = 0.20, Fig. 3).

Patients who received surgery alone without systemic therapy after 1^st^ recurrence had a high relapse rate of 85%. This suggests that surgery alone in the setting of melanoma that has relapsed after adjuvant TT is unlikely to result in long term disease control, and a low threshold to initiate “adjuvant” systemic therapy at this time, or at least careful surveillance, is needed.

We found that the RR to subsequent TT, given as a rechallenge after relapse following adjuvant TT, was 25% (4/16). This is slightly less than the RR seen in other studies examining the efficacy of a TT rechallenge in the stage IV setting; for example, Schreuer et al.^25^ reported a RR of 32% whilst Valpione et al.^26^ reported a RR of 43%. The RR of 25% to a TT rechallenge in our cohort is also lower than the RR of 68% to TT seen in the 1^st^ line metastatic setting.^5^ These results suggest that patients who relapse after adjuvant TT may benefit from a change in treatment modality rather than a rechallenge of TT.

Of the four patients who responded to a rechallenge with TT at first recurrence, 75% (3/4) had developed recurrent melanoma after ceasing adjuvant TT. All four patients had received combination TT as adjuvant therapy. One patient relapsed whilst receiving adjuvant TT and subsequently had a complete response to a rechallenge of TT as 1st line systemic therapy after relapse. However, between relapse and further TT, the patient underwent three surgical resections and adjuvant RT to locally recurrent melanoma. Thus, the interval between cessation of adjuvant TT and rechallenge with TT was two years. These results suggest that maximising the time interval between adjuvant TT and a rechallenge at recurrence may increase the likelihood of subsequent response.

The median time to recurrence from commencement of adjuvant systemic therapy in this cohort was 17.7 months. In patients who relapse after adjuvant anti-PD-1 therapy, median time to recurrence has recently shown to be much shorter, at 4.6 months.^18^ This reflects the fact that recurrences on TT are infrequent, with most recurrences occurring after adjuvant TT is completed. In contrast, recurrences during adjuvant anti-PD-1 therapy are relatively more frequent than recurrence during adjuvant TT, with fewer recurrences occurring after cessation of adjuvant anti-PD-1. For example, Checkmate-238 demonstrated an RFS rate of 70% at the 1-year mark, compared to an RFS rate of 88% at 1 year in the COMBI-AD trial.^13,17^

Limitations of our study include the retrospective nature of data collection, and small sample size. Despite including 21 international sites, we were only able to recruit a modest number of patients, though this is expected given the relative infancy of adjuvant therapy in melanoma and the necessity to include BRAF mutant patients only. Imaging follow-up was performed approximately every 3 months, though not stringently regulated, at each patient’s treatment centre and varied between CT, PET and MRI. Furthermore, measurement of response to subsequent therapy was performed by a patient’s overseeing clinician, rather than a centralised review. Thus, heterogeneity in timing of tumour assessment and response evaluation would have occurred. The exception to this is patients enrolled on a clinical trial, where imaging frequency was stipulated in the respective trial protocol and response assessment occurred via usual Response Evaluation Criteria in Solid Tumours (RECIST) measurements.

## Conclusions

This study suggests that patients who relapse after adjuvant TT respond to subsequent IT-based therapy at comparable rates to the 1st line or treatment naïve setting. Therefore, when considering choice of adjuvant systemic therapy in BRAF mutant melanoma patients, clinicians should consider TT as an option that may not diminish the chance of response to subsequent IT. Furthermore, switching to PD-1 based IT after relapse results in superior RRs and survival than further TT or ipilimumab monotherapy. More evidence is needed to clarify the optimal approach to managing patients with recurrent melanoma after adjuvant TT. Further data on this topic is likely to become available as adjuvant therapy is increasingly utilised and longer follow-up of randomised trials investigating adjuvant TT occurs. Ultimately, decisions about adjuvant therapy for resected stage III/IV BRAF mutant melanoma patients should be made on an individual basis, wherein the choice between adjuvant IT and TT takes into consideration potential toxicities, costs and patient preference.^27^

## Supplementary information

All Supplementary Files

## Data Availability

Data supporting the results reported in this article is stored at Alfred Health and can be provided upon request.
