# Supplementary material for: Melanoma recurrence patterns and management after adjuvant targeted therapy: a multicentre analysis
Source: Br J Cancer. 2020 Oct 22;124(3):574–80. doi: 10.1038/s41416-020-01121-y (PMC7851118; doi:10.1038/s41416-020-01121-y)
Supplement: Supplementary file 1 — All Supplementary Files [file 41416_2020_1121_MOESM1_ESM.docx]

Supplement 1

| **Adjuvant Therapy Characteristics** | **Patient number, N = 85 (%)** |
| --- | --- |
| Adjuvant therapy received- no. (%)  Dabrafenib and Trametinib  Vemurafenib and Cobimetinib  Vemurafenib single agent | 73 (86)  1 (1)  11 (13) |
| Median duration of adjuvant therapy- months (range) | 8.6 (0.2-16.1) |
| Reason for cessation of adjuvant therapy- no. (%)  Completed therapy  Recurrence  Toxicity  Patient choice  Other  Pregnancy  Diagnosis of prostate cancer | 45 (53)  19 (22)  18 (21)  1 (1)  2 (2)  1  1 |

**S1:** Adjuvant therapy received by patients including reason for cessation of therapy.

Supplement 2


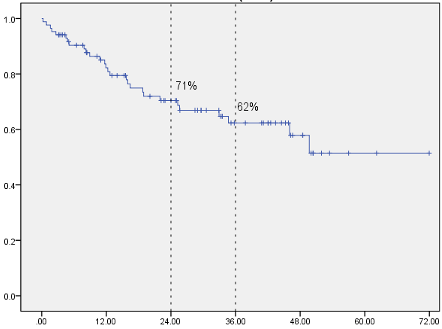


mOS = NR

**Cumulative Survival**

**Time since recurrence (months)**

**Figure S2:** Kaplan-Meier curve of overall survival for all patients from time of first melanoma recurrence. mOS = median overall survival; NR = not reached.

Supplement 3


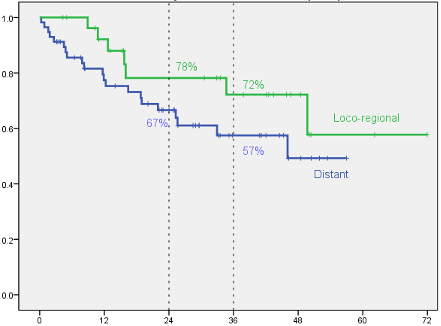


**Cumulative Survival**

**Time since recurrence (months)**

**Figure S3:** Kaplan-Meier curve of overall survival for patients with locoregional or distant relapse at first recurrence after adjuvant TT (*p*=0.16).
